# A randomized phase II trial of 5-fluorouracil, with or without human interferon-β, for advanced colorectal cancer

**DOI:** 10.1038/sj.bjc.6690422

**Published:** 1999-05

**Authors:** A Villar-Grimalt, M T Candel, B Massuti, J Lizón, B Sánchez, A Frau, B Gorostidi, R Goedkoop

**Affiliations:** H. Arnau de Vilanova, Valencia, Spain; H. General de Alicante, Alicante, Spain; H. San Juan, Alicante, Spain; H. Provincial, Castellón, Spain; Laboratorios Serono S.A., Madrid, Spain; Ares-Serono S.A., 15 bis chemin des Mines, Geneva, 1202, Switzerland

**Keywords:** colorectal cancer, 5-fluorouracil, interferon-β, synergism, survival

## Abstract

This study compared the efficacy and safety of 5-fluorouracil (5-FU) monotherapy to that of 5-FU combined with natural human interferon-β (IFN-β) in patients with unresectable, advanced colorectal carcinoma. Forty-nine chemotherapy-naive patients were randomized to 5-FU alone or to the combination. All patients received 750 mg m^−2^ day^−1^ 5-FU for 5 days by continuous intravenous (i.v.) infusion, followed after day 15 by a weekly i.v. bolus of 750 mg m^−2^. IFN-β was injected intramuscularly three times weekly at 9 M IU. Treatment continued for 52 weeks, or until disease progression or intolerable toxicity. Clinical endpoints were tumor response, time to progression, survival and toxicity. The addition of IFN-β to 5-FU significantly improved response rate (33.3% vs 4.5% for evaluable patients; *P* = 0.021), time to progression (median 7.2 vs 4.2 months; *P* = 0.0435), and survival time (median 15.9 vs 7.2 months; *P* = 0.038) without significantly increasing toxicity compared to 5-FU alone. Cumulative 5-FU dose was higher with combined therapy (*P* < 0.001): more patients receiving monotherapy discontinued treatment because of disease progression. Fever was more frequent with combined therapy (*P* = 0.008); there were no other differences in toxicity. The only grade IV toxicity observed was neutropenia (two patients per group). A randomized phase III trial has been initiated to confirm the synergy between 5-FU and IFN-β. © 1999 Cancer Research Campaign


*British Journal of Cancer* (1999) **80**(5/6), 786–791
© 1999 Cancer Research Campaign
Article no. bjoc.1998.0422

### A randomized phase II trial of 5-fluorouracil, with or without human interferon-β, for advanced colorectal cancer

**A Villar-Grimalt[1], MT Candel[1], B Massuti[2], J Lizón[3], B Sánchez[3], A Frau[4], B Gorostidi[5] and R Goedkoop[6]**

[1]H. Arnau de Vilanova, Valencia, Spain; [2]H. General de Alicante, Alicante, Spain; [3]H. San Juan, Alicante, Spain; [4]H. Provincial, Castellón, Spain; [5]Laboratorios Serono S.A., Madrid, Spain; [6]Ares-Serono S.A., 15 bis, chemin des Mines, 1202 Geneva, Switzerland

**Summary** This study compared the efficacy and safety of 5-fluorouracil (5-FU) monotherapy to that of 5-FU combined with natural human interferon-β (IFN-β) in patients with unresectable, advanced colorectal carcinoma. Forty-nine chemotherapy-naive patients were randomized to 5-FU alone or to the combination. All patients received 750 mg $m^{-2}$ $day^{-1}$ 5-FU for 5 days by continuous intravenous (i.v.) infusion, followed after day 15 by a weekly i.v. bolus of 750 mg $m^{-2}$. IFN-β was injected intramuscularly three times weekly at 9 M IU. Treatment continued for 52 weeks, or until disease progression or intolerable toxicity. Clinical endpoints were tumor response, time to progression, survival and toxicity. The addition of IFN-β to 5-FU significantly improved response rate (33.3% vs 4.5% for evaluable patients; $P = 0.021$), time to progression (median 7.2 vs 4.2 months; $P = 0.0435$), and survival time (median 15.9 vs 7.2 months; $P = 0.038$) without significantly increasing toxicity compared to 5-FU alone. Cumulative 5-FU dose was higher with combined therapy ($P < 0.001$): more patients receiving monotherapy discontinued treatment because of disease progression. Fever was more frequent with combined therapy ($P = 0.008$); there were no other differences in toxicity. The only grade IV toxicity observed was neutropenia (two patients per group). A randomized phase III trial has been initiated to confirm the synergy between 5-FU and IFN-β.

**Keywords**: colorectal cancer; 5-fluorouracil; interferon-β; synergism; survival

Colorectal cancer has an annual incidence of 53 cases per 100 000 in the USA and Europe, representing 15% of all malignant tumors (Beard et al, 1995; Netherlands Cancer Registry, 1989). Approximately 30% of patients present with advanced disease, and about 60% of these cases are no longer amenable to surgery. The prognosis for these patients is poor.

The fluorinated pyrimidine 5-fluorouracil (5-FU) has been the principal treatment for advanced colorectal cancer for the past 4 decades (Heidelberger et al, 1957). Therapy with 5-FU leads to objective response in only 8–20% of patients, with few complete responses and minimal improvement in survival (Carter, 1976; Leichman et al, 1995; Moertel, 1982). Dosage of 5-FU is limited by the occurrence of mucosal and neutropenic toxicities (Moertel, 1975).

Various modulating agents have been used in an attempt to synergistically enhance 5-FU's cytotoxicity. Results have so far been disappointing. Improved response rates have been reported with the combination of 5-FU and leucovorin, but responses were mainly partial rather than complete and most were of short duration. Two randomized studies observed significantly increased survival time for 5-FU with leucovorin (Ehrlichman et al, 1988; Poon et al, 1989), but a meta-analysis and two other randomized studies failed to confirm improvement over 5-FU monotherapy (Piedbois et al, 1992; Leichman et al, 1995; O'Dwyer et al, 1996). Early clinical trials reported encouraging results with the combination of 5-FU and interferon alpha (IFN-α), including tumor response rates as high as 35–63% and median survival times of up to 18 months. However, the cost was a high level of toxicity: in particular, fever, constitutional symptoms and myelosuppression (Wadler et al, 1989; Kemeny et al, 1990; Pazdur et al, 1990). Unfortunately, no improvements in response rate or survival time have been observed in further randomized trials of 5-FU with IFN-α versus 5-FU monotherapy (Hill et al, 1995; Greco et al, 1996) or 5-FU with leucovorin (Corfu-A Study Group, 1995).

The interferons are a well-recognized group of naturally occurring proteins with antiviral, immunomodulatory and antiproliferative properties. Based on antigenic specificity, physico-chemical properties and cellular origin, they are classified as type I or type II. The type I interferons, IFN-α and IFN-β, are produced by leucocytes and fibroblasts respectively. IFN-β has a 30% level of amino acid homology with IFN-α (Taniguchi et al, 1980), and binds to the type I interferon receptor with higher affinity than IFN-α (Ruzicka et al, 1987). There is evidence for a receptor-associated protein specifically involved with IFN-β's signalling pathway and not IFN-α's (Platanias et al, 1994). Although the mechanisms of the interferons' anti-tumor activity and 5-FU modulation remain poorly understood, preclinical data suggest a theoretical benefit for IFN-β over IFN-α in the treatment of colorectal cancer. Antiproliferative activity against some tumor cell lines in vitro appears greater for IFN-β than IFN-α (Borden et al, 1982). IFN-β effectively inhibited the growth of several human colon carcinoma cell lines in vitro, in a dose- and

*Received 20 May 1998*
*Revised 29 October 1998*
*Accepted 19 November 1998*

*Correspondence to:* R Goedkoop time-dependent fashion (Guglielmi et al, 1984; Wong et al, 1989). Although IFN-β did not show marked activity against the C-1 colon cancer cell line, addition of IFN-β potentiated 5-FU's effects on C-1 cells in vitro (Kase et al, 1993). IFN-β has been shown to increase in vitro expression of thymidine phosphorylase, thus increasing the sensitivity of human colon carcinoma cells to 5-FU (Schwartz et al, 1995). In a xenograft model (nude mice inoculated with Co-4 colon cancer cells), IFN-β demonstrated dose-dependent anti-tumor effects. In addition, the combination of IFN-β and 5-FU demonstrated increased in vivo anti-tumor effects compared to 5-FU monotherapy, which were not accompanied by enhanced thymidylate synthetase inhibition (Kase et al, 1993). At the inception of the phase II study reported here (protocol GF5909), there were no published data available from clinical trials of combined therapy with IFN-β and 5-FU. The study was therefore undertaken to compare the effects of 5-FU given with natural human IFN-β on response rate, time to disease progression, survival time and safety to those of 5-FU monotherapy in the treatment of patients with unresectable, advanced colorectal carcinoma.

### PATIENTS AND METHODS

### Patient selection

Patients of either sex who met the following criteria were eligible for the study: histologically proven metastatic, locally advanced, or recurrent colorectal carcinoma no longer amenable to surgery and measurable according to World Health Organization (WHO) criteria; age at least 18 years; good Eastern Cooperative Oncology Group performance status (ECOG ≤ 2) and life expectancy of ≥ 3 months; normal cardiac and pulmonary function, and adequate function of the bone marrow (haematocrit ≥ 30%, thrombocytes ≥ 100 l, granulocytes ≥ 1.5 l), liver (bilirubin, alanine aminotransferase, γ glutamyl transferase, and alkaline phosphatase ≤ 1.5 times the upper limit of normal; higher values resulting from known hepatic metastases were allowed) and kidneys (creatinine ≤ 1.5 times the upper limit of normal). Fertile women were required to use effective contraception throughout the study. The following were grounds for exclusion: previous chemotherapy (including 5-FU); treatment with interferons or immunomodulators within the previous year; brain and/or bone metastases as sole localization of the tumor; lesions within previously irradiated fields; other invasive neoplasms; current use of corticosteroids, acetylsalicylic acid, non-steroidal anti-inflammatory drugs (NSAIDs), or barbiturates or other medication interfering with protein synthesis; peptic ulceration; substance abuse; or psychiatric disorders.

Patients gave written informed consent for study participation according to the modified Declaration of Helsinki (1989, Hong Kong). The study was approved by the relevant local Ethics Committees and the Spanish Ministry of Health, and was conducted according to Good Clinical Practice. Patients could be withdrawn from the study for major protocol violations, serious intercurrent illnesses or adverse events, grade IV or persistent grade III toxicities according to the WHO scale, disease progression, or interruption of treatment for more than 2 consecutive weeks for any reason other than dose adjustment for toxicity.

### Treatment plan

This was an open, randomized study conducted in four centers in Spain. Following baseline evaluation, patients who met all eligibility criteria were randomized in a 1:1 ratio to receive either 5-FU monotherapy or the combination of 5-FU and IFN-β for 52 weeks, or until disease progression or the occurrence of intolerable toxicity. Randomization was performed centrally using a table of random numbers, and was not stratified for prognostic factors.

For the first 5 days, all patients received a continuous intravenous (i.v.) infusion of 5-FU at a dose of 750 mg $m^{-2}$ per day. From day 15 until the end of the study, all patients received weekly i.v. bolus injections of 750 mg $m^{-2}$ 5-FU, and patients randomized to combination treatment received human fibroblast-derived IFN-β (Frone®, Laboratorios Serono S.A., Madrid, Spain) as intramuscular (i.m.) injections of 9 M IU three times a week. This schedule, previously reported for the combination of IFN-α and 5-FU (Wadler et al, 1989), has been used in other randomized studies of IFNs with 5-FU.

Toxicity was assessed according to the modified WHO recommendations for grading of acute and subacute toxicities (World Health Organization, 1979). Dose modifications were made for particular toxicities known to be associated with the study drugs: for 5-FU, the dose could be reduced by 33% until the toxicity resolved, or treatment could be interrupted for up to 2 weeks, to be resumed at the reduced dose if toxicity resolved. It was recommended that paracetamol be given for IFN-β-related side-effects such as fever or constitutional symptoms; however, for specified grade II or III toxicities the IFN-β dose was reduced by 33% until resolution. In all cases, a maximum of 4 weeks was allowed for resolution: if this did not occur, or if the toxicity recurred, the patient was withdrawn from the study.

During the study paracetamol, NSAIDs, codeine and morphic agents were allowed for symptomatic use only. Other medications considered necessary for a patient's welfare that would not interfere with study medication or assessment were given at the investigator's discretion.

Following prestudy assessment, patients were monitored at weekly intervals through physical examination and assessment of body weight and hematological parameters. Biochemical analysis was performed monthly. Carcinoembryonic antigen (CEA) levels, patient performance status (ECOG) and tumor response were measured at 8-weekly intervals. All assessments were performed until treatment discontinuation.

### Response criteria

The main efficacy variable, tumor response, was evaluated every 8 weeks using computerized tomography (CT) scan, X-ray, or ultrasound, and was assessed according to WHO response criteria (World Health Organization, 1979). Clinically measurable disease consisted of bidimensionally measurable lesions with clearly defined margins on X-ray, CT scan, or ultrasound. Lesions serving as measurable disease had to be at least 1 cm × 1 cm in size. Complete response (CR) was defined as disappearance of all known lesions lasting for at least 4 weeks. Partial response (PR) referred to decrease by at least 50% in total tumor mass size, lasting for at least 4 weeks, without occurrence of new lesions. No change (NC) referred to a decrease in tumor mass size of less than 50%, or an increase of less than 25% determined on two occasions at least 8 weeks apart, without new lesions. Progression of disease (PD) was defined as an increase in tumor mass size of more than 25% or appearance of a new lesion. Secondary endpoints were time to progression, defined as time between initiation of treatment and first observation of PD, and survival time, defined as

© Cancer Research Campaign 1999

time between initiation of treatment and death or final analysis (whichever came first). The main criterion for safety assessment was occurrence of treatment-emergent toxicity.

### Statistical methodology

Sample size was not calculated using statistical methodology because this was a pilot study. It was assumed that 20 evaluable patients per treatment arm would allow assessment of response rates; therefore 51 patients were enrolled. Continuous variables were assessed using Student's *t*-tests; categorical variables, including tumor response and safety measurements, were assessed using the $\chi^2$ or Fisher's exact test as appropriate. Total time on treatment and dose reductions for either drug were recorded prospectively. Curves for overall survival time were calculated using the Kaplan–Meier method, with results expressed as the median with 95% confidence intervals. Distributions of survival time and time to progression for the two treatments were compared using the generalized Wilcoxon test (called 'Breslow' in SPSS statistical software).

Two populations were analysed for efficacy. The intent-to-treat population included all eligible and randomized patients. The evaluable patient population included all randomized patients without major protocol violations who underwent at least one assessment of tumor response. All randomized patients were included in the safety analysis, which was performed using the Fisher's exact and $\chi^2$ tests.

### RESULTS

### Patients

From 8 March 1993 to 25 November 1994, a total of 51 patients were randomized to receive 5-FU monotherapy ($n$ = 26) or 5-FU–IFN-β combination therapy ($n$ = 25). Two randomized patients in the 5-FU group were found ineligible (one patient was using an NSAID at enrollment and the other had non-measurable disease) and were therefore excluded from the intent-to-treat population ($n$ = 49). A further six patients, two from the 5-FU group and four from the 5-FU–IFN-β group, were excluded from the evaluable patient population ($n$ = 43) because of major protocol violations or lack of assessments of response.

Demographic and baseline characteristics are shown in Table 1. No baseline variables differed significantly between treatment groups. The most common site of measurable lesions was the liver (74.1% and 64.3% of patients in the 5-FU and 5-FU–IFN-β groups respectively), with the lungs being the next most frequent location (14.8% and 17.9% of patients respectively).

### Treatment dosage and duration

The mean weekly 5-FU dose was significantly higher for the 5-FU monotherapy group (1082 mg) than for the 5-FU–IFN-β combination therapy group (935 mg; $P$ = 0.022, *t*-test). The mean 5-FU dose intensity was also significantly higher for the 5-FU group (82.9% vs 73.5%; $P$ = 0.015, *t*-test).

No patients in the 5-FU group completed the planned treatment period, compared to four patients (16%) in the 5-FU–IFN-β group. The mean treatment duration for the 5-FU–IFN-β group, at 30.4 weeks, was nearly double that for the 5-FU group (13.6 weeks; $P$ < 0.001, *t*-test), and the cumulative dose of 5-FU was significantly higher in the 5-FU–IFN-β group (29.527 vs 15.394 g; $P$ = 0.001, *t*-test). The mean weekly dose of IFN-β administered (22.3 ± 5.6 M IU per week) was close to the proposed dose of 27 M IU per week.

The 5-FU dose was modified for 88% of patients in the 5-FU group and 92% in the 5-FU–IFN-β group. The proportion of patients requiring both 5-FU dose reduction and treatment interruption was higher in the 5-FU–IFN-β group (82.6% vs 52.4%; $P$ = 0.02, Fisher). The IFN-β dose was adjusted in 84% of patients receiving combined treatment.

**Table 1** Patient baseline characteristics

| Eligible and randomized patients | 5-FU ($n$ = 24) | | 5-FU–IFN-β ($n$ = 25) | |
|---|---|---|---|---|
| | No. | % | No. | % |
| Sex (% female) | | 46 | | 40 |
| Age in years (mean ± SD) | 62.0 (± 7.6) | | 65.2 (± 6.7) | |
| Weight in kg (mean ± SD) | 70.9 (± 9.6) | | 65.4 (± 12.4) | |
| ECOG performance status | | | | |
| 0–1 | 17 | 70.8 | 23 | 92 |
| 2 | 7 | 29.2 | 2 | 8 |
| Diagnosis of disease | | | | |
| Locally advanced | 0 | 0 | 0 | 0 |
| Metastatic | 22 | 91.6 | 18 | 72 |
| Recurrent | 1 | 4.2 | 4 | 16 |
| Local and metastatic | 0 | 0 | 1 | 4 |
| Metastatic and recurrent | 1 | 4.2 | 2 | 8 |
| Primary tumour site | | | | |
| Ascending colon | 4 | 16.7 | 4 | 16 |
| Descending colon | 2 | 8.3 | 3 | 12 |
| Sigmoid colon | 2 | 8.3 | 7 | 28 |
| Rectum–sigmoid colon | 5 | 20.8 | 4 | 16 |
| Rectum | 10 | 41.7 | 5 | 20 |
| Undefined colon | 1 | 4.2 | 2 | 8 |
| Histological grade | | | | |
| Gx | 6 | 25 | 9 | 36 |
| G1 | 1 | 4.2 | 3 | 12 |
| G2 | 16 | 66.7 | 11 | 44 |
| G3 | 1 | 4.2 | 2 | 8 |
| Dukes' stage | | | | |
| A | 2 | 8.3 | 2 | 8 |
| B | 2 | 8.3 | 0 | 0 |
| C | 6 | 25 | 4 | 16 |
| D | 14 | 58.3 | 17 | 68 |
| Unknown | 0 | 0 | 2 | 8 |
| Measurable lesions | | | | |
| Pelvic mass | 1 | 3.7 | 3 | 10.7 |
| Liver | 20 | 74.1 | 18 | 64.3 |
| Lung | 4 | 14.8 | 5 | 17.9 |
| Suprarenal | 1 | 3.7 | 0 | 0 |
| Adenopathies | 0 | 0 | 1 | 3.6 |
| Retroperitoneal | 1 | 3.7 | 1 | 3.6 |
| CEA in ng ml$^{-1}$ (mean ± s.d.) | | | | |
| | 729 (± 1544) | | 279 (± 824) | |

### Efficacy

Results are presented for the intent-to-treat population ($n$ = 49). Objective tumor response is also presented for evaluable patients ($n$ = 43).

Among evaluable patients, an overall tumor response rate of 4.5% was observed in the 5-FU group (one PR), compared with a rate of 33.3% in the 5-FU–IFN-β group (two CR and five PR; $P$ = 0.021, Fisher). The majority of patients in both groups showed no change (12 of 22 in the 5-FU and 11 of 21 in the 5-FU–IFN-β groups).

© Cancer Research Campaign 1999

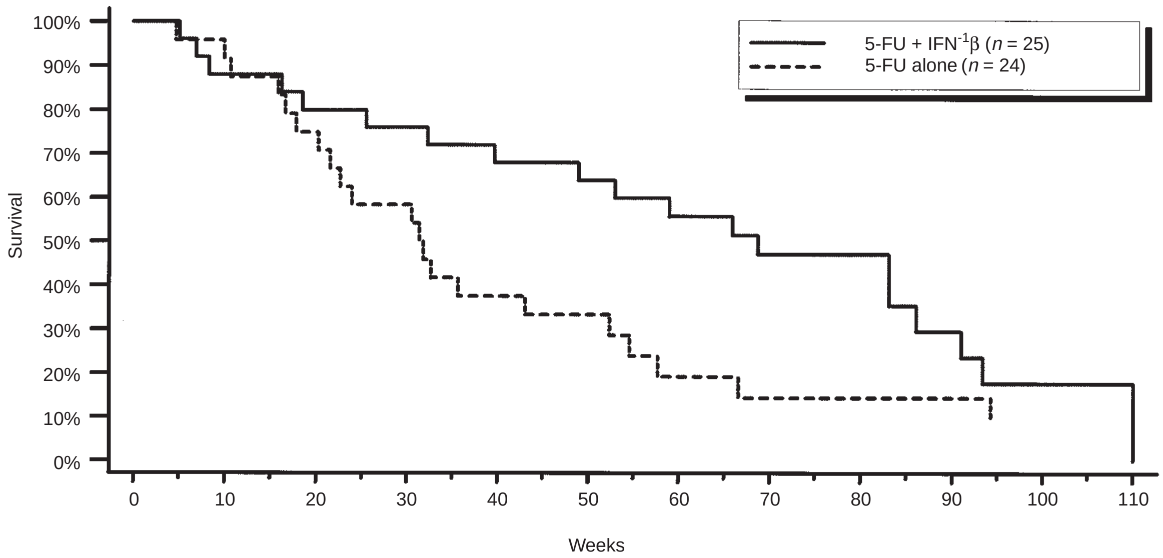

**Figure 1** Survival time: Kaplan–Meier estimates of survival function for the intent-to-treat population. A statistically significant increase in survival occurred with the addition of IFN-β to 5-FU (Breslow, $P = 0.038$)

**Table 2** Haematological and non-haematological toxicity

| | Grade I–II toxicity | | Grade III–V toxicity | |
|---|---|---|---|---|
| | 5-FU ($n = 26$) | 5-FU–IFN-β ($n = 25$) | 5-FU ($n = 26$) | 5-FU–IFN-β ($n = 25$) |
| Haemoglobin | 10 | 9 | 2 | 2 |
| Neutrophils | 9 | 11 | 6 | 6 |
| Thrombocytes | 6 | 4 | 0 | 0 |
| Diarrhoea | 12 | 17 | 3 | 2 |
| Mucositis/stomatitis | 13 | 14 | 3 | 4 |
| Vomiting | 19 | 12 | 1 | 4 |
| Conjunctivitis | 4 | 8 | 1 | 0 |
| Cutaneous toxicity | 8 | 6 | 2 | 2 |
| Fever* | 8 | 15 | 0 | 2 |
| Lethargy | 2 | 6 | 0 | 1 |
| Infection | 1 | 2 | 1 | 1 |
| Alopecia | 2 | 4 | 0 | 1 |
| Ataxia | 4 | 4 | 0 | 1 |
| Neurological toxicity | 9 | 12 | 2 | 3 |

No significant differences were observed for toxicity, except for grouped grade I + II versus grade III + IV fever* (Fisher, $P = 0.016$).

Intent-to-treat analysis of tumor response rate supported this significant finding (response rates 4.17% and 28% for 5-FU and 5-FU–IFN-β respectively; $P = 0.0488$). In the intent-to-treat population, median time to disease progression was significantly shorter in the 5-FU group (4.2 months; 95% CI 2.7–5.4) than in the 5-FU–IFN-β group (7.2 months; 95% CI 6.1–8.2; $P = 0.0435$, Breslow).

Figure 1 shows the Kaplan–Meier estimates of survival function for time to death (from any cause) in the intent-to-treat population. Median survival time was significantly shorter in the 5-FU group (7.2 months; 95% CI 4.8–9.7) than in the 5-FU–IFN-β group (15.9 months; 95% CI 10.4–21.3; $P = 0.038$, Breslow). At 12 months, six patients in the 5-FU group (27.3%) and 14 patients in the 5-FU–IFN-β group (66.7%) were still alive. Rescue treatment was given, after discontinuation of the study treatment, to seven patients in 5-FU group (29.2%) and to nine patients in the 5-FU–IFN-β group (36%) ($P = 0.84$, $\chi^2$).

### Tolerability and safety

Toxicities are presented by WHO grade in Table 2. For each category, each patient is represented by the highest-grade toxicity experienced. Two patients from each treatment group experienced grade IV neutropenia; no other grade IV toxicities were observed. Few patients experienced clinically significant hematological toxicity (≥ grade I), as reflected by weekly assessment of hemoglobin level and neutrophil and platelet counts.

The only statistically significant difference between the groups was a higher frequency of fever in the 5-FU–IFN-β group (68% vs 31%; $P = 0.008$, $\chi^2$). Two patients who received 5-FU–IFN-β experienced grade III fever. Three cases of grade III diarrhea occurred in the 5-FU and two in the 5-FU–IFN-β groups ($P = 0.27$, Fisher). Grade III mucositis/stomatitis requiring interruption of 5-FU occurred in three patients in the 5-FU and four in the 5-FU–IFN-β groups ($P = 0.67$, Fisher). Grade II/III skin toxicity requiring 5-FU dose reduction occurred in four and six patients in the 5-FU and 5-FU–IFN-β groups respectively ($P = 0.46$, Fisher), and grade II or higher neurological toxicity was observed in eight and seven patients respectively ($P = 0.29$, Fisher).

### DISCUSSION

Compared to published results concerning modulation of 5-FU by IFN-α in treatment of advanced colorectal cancer, clinical data on IFN-β in this indication are limited. Nevertheless, IFN-β compares favorably with IFN-α in its capacity to inhibit the growth of colon cancer cells (Guglielmi et al, 1984; Wong et al, 1989), to modulate 5-FU in vitro (Kase et al, 1993), and to express receptor and post-receptor signal transduction activity (Platanias et al, 1994). The combination of 5-FU and IFN-β was therefore of prime interest.

© Cancer Research Campaign 1999

This randomized phase II trial studied the effect of 5-FU–IFN-β on tumor response in comparison with 5-FU monotherapy in chemotherapy-naive patients with unresectable, advanced colorectal cancer. The two treatment groups were well balanced (Table 1): Dukes' D disease was more common in the 5-FU–IFN-β group, and more patients in the 5-FU group had poor performance status (ECOG 2); these differences were not statistically significant.

The tumor response rate among evaluable patients in this study was 33.3% in the 5-FU–IFN-β group; only one patient receiving monotherapy (4.5%) showed objective response. Intent-to-treat analysis supported this finding (tumor response rates 28% and 4.17% for combined therapy and monotherapy respectively). Although any comparison of efficacy between studies requires caution, the response rate seen with 5-FU monotherapy appears low compared to results of other randomized trials using the same 5-FU schedule (Hill et al, 1995; Dufour et al, 1996; Greco et al, 1996). The response rate seen with 5-FU–IFN-β is consistent with results from the only published study to use 5-FU with IFN-β in chemotherapy-naive patients with advanced colorectal cancer (35%; Wadler et al, 1995) and from randomized studies of 5-FU with IFN-α that used the same 5-FU schedule (Corfu-A Study Group, 1995; Hill et al, 1995; Dufour et al, 1996; Greco et al, 1996; Jäger et al, 1996): these studies reported response rates between 21% and 30%. Randomized trials of 5-FU with leucovorin, using different 5-FU schedules, reported similar rates (18–39%; Piedbois et al, 1992; Corfu-A Study Group, 1995; Köhne et al, 1995; Leichman et al, 1995; Jäger et al, 1996).

In the present study, intent-to-treat analysis showed a 71% increase in median time to disease progression for 5-FU–IFN-β compared to monotherapy (7.2 vs 4.2 months). Median survival was also significantly increased by the addition of IFN-β to 5-FU: in the intent-to-treat analysis, from 7.2 to 15.9 months. The patients who received rescue treatment (high-dose continuous i.v. infusion of 5-FU, UFT with or without leucovorin) after discontinuation of study treatment were equally distributed between the two groups. The median survival time reported for patients receiving 5-FU with either IFN-α or leucovorin approximates 12 months. Combination of 5-FU with recombinant IFN-β showed a median survival time of 15 months (Wadler et al, 1995). To date, only one randomized study of 5-FU with IFN-α has reported significant improvement in survival time compared to monotherapy (Dufour et al, 1996); three others have observed no significant benefit (Hill et al, 1995; Greco et al, 1996; O'Dwyer et al, 1996). Although two studies showed significant increases in survival time using leucovorin with 5-FU (Ehrlichman et al, 1988; Poon et al, 1989), a meta-analysis including these studies and seven others (Piedbois et al, 1992) could not confirm improvement over 5-FU monotherapy. In addition, a multi-armed randomized study using different 5-FU regimens with or without leucovorin failed to demonstrate differences in survival between 5-FU with leucovorin and 5-FU only (Leichman et al, 1995).

Improved efficacy must always be weighed against treatment-emergent toxicities. In the current study, the only significant difference in toxicity between the two treatments was a higher occurrence of fever with 5-FU–IFN-β. Grade III fever occurred in two patients receiving 5-FU–IFN-β; no grade IV fever occurred. Patients received paracetamol for flu-like symptoms, which are well-recognized side-effects of interferon therapy and are generally transient and self-limiting. Treatment with 5-FU–IFN-β was not associated with more neurological toxicity, myelosuppression, or gastrointestinal toxicity than 5-FU monotherapy. No grade IV toxicities were observed, with the exception of two cases of neutropenia in each treatment group. No patients were withdrawn from the study or hospitalized because of adverse events.

The combination of 5-FU and IFN-β may be less toxic than 5-FU with IFN-α. Combinations of 5-FU with IFN-α and with leucovorin were compared in a randomized trial using the same 5-FU schedule as the present study for the 5-FU–IFN-α group (Corfu-A Study Group, 1995). With the caution necessary when comparing different studies, it would appear that combined grade III and IV hematological toxicity was less common with IFN-β than with IFN-α, particularly with regard to neutropenia (24% vs 74%). Occurrences of grade III and IV mucositis/stomatitis and vomiting were similar for the two interferons, while diarrhea was more frequent with 5-FU–IFN-α than with 5-FU–IFN-β (35% and 8% respectively). The mean cumulative 5-FU dose was significantly lower in the monotherapy group than in the 5-FU–IFN-β group: more patients in the monotherapy group discontinued treatment because of disease progression. Approximately 90% of patients in both groups required 5-FU dose adjustments for toxicity. The IFN-β dose was also adjusted in the majority of patients receiving combined treatment.

In conclusion, this randomized pilot study demonstrated a synergistic anti-tumor action for 5-FU and natural IFN-β in the treatment of chemotherapy-naive patients with advanced colorectal cancer. The combination resulted in both increased tumor response and improved survival, without higher levels of clinically relevant drug-related toxicity. The toxicity profile of IFN-β plus 5-FU may compare favorably to that of 5-FU and IFN-α, suggesting that 5-FU–IFN-β may be a promising alternative for the treatment of advanced colorectal cancer. A multicenter, randomized phase III trial comparing 5-FU–IFN-β to standard treatment has been initiated recently.

### ACKNOWLEDGEMENTS

We gratefully acknowledge the collaboration and commitment of the investigators and their staff, Ignacio Alvarez (biostatistician, Laboratorios Serono S.A., Madrid, Spain), and Florilène Dupont (biostatistician, Ares-Serono S.A., Geneva, Switzerland), without whom the study would not have been possible, and the assistance of Annette Dubois (medical writer, Ares-Serono S.A., Geneva, Switzerland) in the preparation of the manuscript.

### REFERENCES

Beard CM, Spencer CJ, Weiland LH, O'Fallon WM and Melton LJ 3rd (1995) Trends in colorectal cancer over half a century in Rochester, Minnesota, 1940–1989. *Ann Epidemiol* **5**: 210–214

Borden EC, Hogan TF and Voelkel JG (1982) Comparative antiproliferative activity in vitro of natural interferons alpha or beta for diploid and transformed human cells. *Cancer Res* **42**: 4989–4953

Carter SK (1976) Large bowel cancer: The current status of treatment. *J Natl Cancer Inst* **56**: 3–10

Corfu-A Study Group (1995) A phase III randomised study of two fluorouracil combinations of either interferon α 2a or leucovorin for advanced colorectal cancer. *J Clin Oncol* **13**: 921–928

Dufour P, Husseini F, Dreyfus B, Cure H, Martin C, Prevost G, Olivier JP, Dumas F, Duclos B, Olivares R, Leszler A, Bergerat JP, Audhuy B, Thill L and Oberling F (1996) 5-Fluorouracil versus 5-fluorouracil plus α-interferon as treatment of metastatic colorectal carcinoma. A randomized study. *Ann Oncol* **7**: 575–579

 *© Cancer Research Campaign 1999*

Ehrlichman C, Fine S, Wong A and Elhakim T (1988) A randomized trial of fluorouracil and folinic acid in patients with metastatic colorectal carcinoma. *J Clin Oncol* **6**: 469–475

Greco J, Figlin R, York M, Einhorn L, Schilsky R, Marshall EM, Buys SS, Froimtchuk MJ, Schuller J, Schuchter L, Buyse M, Ritter L, Man A and Yap AK (1996) Phase III randomized study to compare interferon α-2a in combination with fluorouracil versus fluorouracil alone in patients with advanced colorectal cancer. *J Clin Oncol* **14**: 2674–2681

Gugliemi A, Mori A, Aschele C, Rosso R and Sobrero A (1984) 5-Fluorouracil plus beta interferon against human colon carcinoma cells in vitro. *J Interferon Res* **12**: S168

Heidelberger C, Chaudhari NK, Danneberg P, et al (1957) Fluorinated pyrimidines. A new class of tumour-inhibitory compounds. *Nature* **179**: 663–666

Hill M, Norman A, Cunningham D, Findlay M, Nicholson V, Hill A, Iveson A, Evans C, Joffe J, Nicholson M, et al (1995) Royal Marsden phase III trial of 5-fluorouracil with or without interferon alfa 2b in advanced colorectal cancer. *J Clin Oncol* **13**: 1297–1302

Jäger E, Heike M, Bernard H, Klein O, Bernhard G, Lautz D, Michaelis J, Meyer zum Bushenfelde KH and Knuth A (1996) Weekly combined high-dose leucovorin versus low-dose leucovorin combined with fluorouracil in advanced colorectal cancer: results of a randomized multicenter trial. *J Clin Oncol* **14**: 2274–2279

Kase S, Kubota T, Watanabe M, Furukawa T, Tanino H, Ishibiki K, Teramoto T and Kitajima M (1993) Interferon beta increases antitumour activity of 5-fluorouracil against human colon carcinoma cell lines in vitro and in vivo. *Anticancer Res* **13**: 69–74

Kemeny N, Younes A, Seiter K, Kelsen D, Sammarco P, Adams L, Derby S, Murray P and Houston C (1990) Interferon alpha-2a and 5-fluorouracil for advanced colorectal carcinoma. Assessment of activity and toxicity. *Cancer* **66**: 2470–2475

Köhne CH, Wilke H, Hecker H, Schoffski P, Kaufer C, Rauschecker H, Andreesen R, Ohl U, Lange HJ, Klaassen U, et al (1995) Interferon-alpha does not improve the antineoplastic efficacy of high-dose infusional 5-fluorouracil plus folinic acid in advanced colorectal cancer. *Ann Oncol* **6**: 461–466

Leichman CG, Fleming TR, Muggia FM, Tangen CM, Ardalan B, Doroshow JH, Meyers FJ, Holcombe RF, Weiss GR, Mangalik A and Macdonald JS (1995) Phase II study of fluorouracil and its modulation in advanced colorectal cancer: a Southwest Oncology Group study. *J Clin Oncol* **13**: 1303–1311

Moertel CG (1975) Clinical management of advanced gastrointestinal cancer. *Cancer* **36**: 675–682

Moertel CG (1982) Large bowel. In *Cancer Medicine*, 2nd edn, Holland JF, Frei E (eds), pp. 830–1859. Lea & Febiger: Philadelphia, PA

Netherlands Cancer Registry (1989) *Incidence of Cancer in the Netherlands*. First Report of the Netherlands Cancer Registry, pp. 36–42

O'Dwyer PJ, Ryan LM, Valone FH, Hines JD, Arbuck SG, Wadler S, Haller DG, Mayer RJ and Benson AB (1996) Phase III trial of biochemical modulation of 5-fluoruracil by iv or oral leucovorin or by interferon in advanced colorectal cancer: an ECOG/CALBG phase III trial. *Proc ASCO* **15**: 207 (abstract 469)

Pazdur R, Ajani JA, Patt YZ, Winn R, Jackson D, Shepard B, DuBrow R, Campos L, Quaraishi M, Faintuch J, et al (1990) Phase III study of fluorouracil and recombinant interferon α-2a in previously untreated advanced colorectal carcinoma. *J Clin Oncol* **8**: 2027–2031

Piedbois P, Buyse M, Rustum Y, Machover D, Erlichman C, Carlson RW, Valone F, Labianca R, Doroshow JH and Petrelli (1992) Modulation of fluorouracil by leucovorin in patients with advanced colorectal cancer: evidence in terms of response rate. *J Clin Oncol* **10**: 896–903

Platanias LC, Uddin S and Colamonici OR (1994) Tyrosine phosphorylation of A and B subunits of type I interferon receptor. *J Biol Chem* **269**: 17761–17764

Poon MA, O'Connell MJ, Moertel CG, Wieand HS, Cullinan SA, Everson LK, Krrok JE, Maillard JA, Laurie JA, Tschetter LK, et al (1989) Biochemical modulation of fluorouracil: evidence of significant improvement of survival time and quality of life in patients with advanced colorectal carcinoma. *J Clin Oncol* **7**: 1407–1418

Ruzicka FJ, Jach ME and Borden EC (1987) Binding of recombinant-produced interferon-beta-ser to human lymphoblastoid cells. *J Biol Chem* **269**: 17761–17764

Schwartz EL, Baptiste N, Wadler S and Makower D (1995) Thymidine phosphorylase mediates the sensitivity of human colon carcinoma cells to 5-fluorouracil. *J Biol Chem* **270**: 19073–19077

Taniguchi T, Manei N, Schwarzstein M, Nagata S, Muramatsu M and Weissman C (1980) Human leukocyte and fibroblast interferons are structurally related. *Nature* **285**: 547–549

Wadler S, Schwartz EL, Goldman M, Lyver A, Rader M, Zimmerman M, Itri L, Weinberg V and Wiernick PH (1989) Fluorouracil and recombinant alfa-2a-interferon: an active regimen against advanced colorectal carcinoma. *J Clin Oncol* **7**: 1769–1775

Wadler S, Haynes H, Tenteromano L, Kaleya R and Rozenblit PH (1995) Results of sequential phase II clinical trials of 5-fluorouracil (5FU) + interferon-beta (IFNβ) in patients with advanced colorectal cancer. *Proc ASCO* **14**: 205 (abstract 499)

Wong VYL, Rieman DJ, Aronson L, Dalton BJ, Greig R and Anzano MA (1989) Growth inhibitory activity of interferon-beta against human colorectal carcinoma cell lines. *Int J Cancer* **43**: 526–530

World Health Organization (1979) *Handbook for Reporting Results of Cancer Treatment*. WHO Offset Publication no. 48, World Health Organization: Geneva, Switzerland

*© Cancer Research Campaign 1999*
